# Sexual Dimorphism in Transcriptional and Functional Glucocorticoid Effects on Mouse Skeletal Muscle

**DOI:** 10.3389/fendo.2022.907908

**Published:** 2022-07-11

**Authors:** Sheng Li, Milena Schönke, Jacobus C. Buurstede, Tijmen J.A. Moll, Max Gentenaar, Maaike Schilperoort, Jenny A. Visser, Kasiphak Kaikaew, Davy van de Vijver, Tooba Abbassi-Daloii, Vered Raz, Annemieke Aartsma-Rus, Maaike van Putten, Onno C. Meijer, Jan Kroon

**Affiliations:** ^1^ Department of Medicine, Division of Endocrinology, Leiden University Medical Center, Leiden, Netherlands; ^2^ Einthoven Laboratory for Experimental Vascular Medicine, Leiden University Medical Center, Leiden, Netherlands; ^3^ Department of Internal Medicine, Erasmus MC, University Medical Center Rotterdam, Rotterdam, Netherlands; ^4^ Department of Physiology, Faculty of Medicine, Chulalongkorn University, Bangkok, Thailand; ^5^ Department of Human Genetics, Leiden University Medical Center, Leiden, Netherlands

**Keywords:** androgen, betamethasone, corticosterone, glucocorticoids, muscle atrophy

## Abstract

Muscle atrophy is common in patients with increased glucocorticoid exposure. Glucocorticoid effects are often sex-specific, and while different glucocorticoid responses between male and female subjects are reported, it is unclear why this is. In this study, we evaluated the effects of corticosterone and synthetic glucocorticoid treatment on muscle atrophy in male and female mice. We found that corticosterone treatment reduced grip strength in female mice only, whereas muscle mass was reduced in both sexes. Skeletal muscle transcriptional responses to corticosterone treatment were more pronounced and widespread in male mice. Synthetic glucocorticoid treatment reduced grip strength in both sexes, while female mice were more sensitive to muscle atrophy than male mice. To evaluate the role of androgens, chemically-castrated male mice were treated with synthetic glucocorticoids. We observed additively reduced muscle mass, but did not observe any interaction effects. Although sex differences in glucocorticoid responses in skeletal muscle are partly influenced by androgen signaling, further studies are warranted to fully delineate the underlying mechanisms.

## Introduction

Muscle atrophy is the wasting or loss of muscle tissue and is observed in multiple diseases including cancer, diabetes, sepsis and renal failure, but also upon synthetic glucocorticoid (GC) treatment. In the elderly and aforementioned patients, muscle atrophy significantly reduces the quality of life and increases mortality (1–3). GC-induced muscle atrophy is prevalent, and is mostly the result of a high dose and sustained usage of GCs or chronic increased endogenous GC levels (4, 5). Indeed, muscle atrophy and reduced muscle function are observed at different doses and treatment regimens with synthetic glucocorticoids such as dexamethasone and prednisolone (6–9). Another commonly used synthetic glucocorticoid is betamethasone (10), which is prescribed for a range of inflammatory diseases at a wide range of doses (11).

Skeletal muscle is composed of different muscle fiber types, including the slow/oxidative type 1 fibers and fast/glycolytic type 2 fibers (12, 13). Type 1 fibers have an oxidative capacity and contain more myoglobin and mitochondria. These fibers are primarily important for muscle endurance, and have a higher resistance to fatigue and reduced force generation compared to type 2 fibers (14). Type 2 fibers are predominantly glycolytic and can be subdivided in several subtypes including type 2A, 2X and 2B fibers. Type 2A fibers have a fast contraction velocity and are less prone to fatigue compared to type 2B fibers. Type 2B fibers are the largest fiber type and generate ATP by anaerobic metabolic processes when maximum power is required (15). Skeletal muscles are composed of distinct mixtures of fiber types (16), and changes in muscle function and atrophy often involve a re-distribution in muscle fiber type composition (17).

Total muscle mass is regulated by many endocrine factors, including anabolic factors such as androgens and catabolic factors such as GCs (18, 19). GCs negatively regulate muscle mass both directly and indirectly. Direct effects include the upregulation of atrophy-related factors, and indirect effects are mediated e.g. *via* interference with anabolic pathways. Altogether this results in a reduction of protein and muscle fiber number and density (20, 21). Upon GC exposure in skeletal muscle, the ubiquitin-proteasome system is activated, which plays a major role in myofibrillar protein degradation (22). Muscle atrophy F‐box (*atrogin‐1*) and muscle ring finger 1 (*MurF-1*) are two muscle-specific ubiquitin ligases of which expression is increased under atrophy-inducing conditions, and these so-called atrogenes play a critical role in muscle atrophy (23, 24). Krüppel-like transcription factor (*Klf15*) is a pivotal factor in skeletal muscle, and was shown to directly regulate the expression of the *atrogin-1* and *MurF-1* atrogenes (25) but is also involved in muscle endurance (26).

Many processes that are influenced by GC exposure are known to be sexually dimorphic (27, 28), oftentimes due to differences in sex hormone levels. Synthetic glucocorticoid treatment influences sex hormone levels, i.e. it lowers the testosterone level in male rats, while glucocorticoid treatment increases testosterone in female rats (29). In humans, glucocorticoids response in muscle function can be sexually dimorphic, but it is unclear to what extent androgens play a role in such effects. In this study, we investigated the effects of corticosterone and synthetic glucocorticoid treatment on muscle atrophy and function in male and female mice. We found that male and female muscle responded differently to glucocorticoid exposure at a transcriptomic and functional level, and that androgen signaling may in part contribute to these differences.

## Methods

### Animals

All animal experiments were approved by the ethical committee of Leiden University Medical Center (functional cohorts) or Erasmus MC (RNA-sequencing cohort). Mice were purchased from Charles Rivers Laboratories and group housed in conventional cages with a 12-hour:12-hour light:dark cycle and had *ad libitum* access to water and RM3 chow diet (Special Diet Services, Essex, UK). Male and female C57BL/6J mice aged 8-10 weeks were used.

### Animal Experiments

To test muscle sensitivity to corticosterone treatment, male (N=8/group) and female mice (N=8/group) were implanted subcutaneously with either a corticosterone-pellet (20 mg corticosterone and 80 mg cholesterol) or a vehicle-pellet (100 mg cholesterol) in the neck region (30, 31), and mice were followed for 14 days. Corticosterone and vehicle pellets were synthesized at Leiden University Medical Center.

To study sex differences in sensitivity to synthetic glucocorticoid betamethasone treatment, male (N=4/group) and female mice (N=6/group) were intraperitoneally injected with 3 mg/kg betamethasone, 25 mg/kg betamethasone, or PBS (vehicle) daily for 14 days. The dose of betamethasone was based on previous muscle atrophy studies with dexamethasone (32), which has approximately the same potency as compared to betamethasone.

To investigate the role of androgen signaling in glucocorticoid-induced muscle atrophy, male mice were chemically castrated using a subcutaneous injection with 25 mg/kg degarelix (MedChemExpress), which is a GnRH antagonist that blocks LH and FSH release and results in diminished testosterone levels (33). Intact and chemically-castrated mice were intraperitoneally injected daily with 3 mg/kg betamethasone or vehicle (PBS) for 14 days (N=8/group).

### Body Weight, Body Composition, Grip Strength and Grid Hanging Measurement

All cohorts were subjected to several functional tests and measurements to assess body weight, body composition and muscle function. Body weight, body composition (EchoMRI-100-analyzer) and grip strength were measured twice a week and grid hanging was measured once a week, and all functional measurements were performed between 3-6 hours after lights-on. Grip strength of the forelimb was measured using a grid attached to an isometric force transducer (Chatillon, Columbus Instruments 080529). The force transducer records the maximum force that is required to break the mouse’s grip from the mesh surface. In total, we recorded five sets of measurements, each consisting of three pulls and with a resting period of at least one minute between them. The three highest values obtained were averaged. Overall muscle function was assessed with the four limbs hanging test, the mouse was placed on a grid, which was turned upside down, 15 cm above a cage filled with soft bedding. This test was performed weekly with a maximum of three attempts per session from which the best performance was used. Maximum allowed hanging time was 600 seconds. At the end of the experiments, mice were killed by CO_2_ asphyxiation (between 3-6 hours after lights-on) and several muscle types were isolated, weighed and frozen in liquid nitrogen for further processing.

### RNA Isolation and RT-qPCR Analysis

Total RNA was isolated by using Tripure (Roche) according to the manufacturer’s instructions. RNA concentration was measured by NanoDrop spectrophotometer (Thermo Fisher). Total RNA was diluted into 1 µg for reverse transcription using M-MLV reverse-transcriptase (Promega). cDNA (4 ng) was used per 10 µl RT-qPCR reaction, and each qPCR reaction contained 1 µl primers (0.5 µl forward and 0.5 µl reverse of each) and 5 µl SYBR green supermix (Bio-Rad) using a Bio-Rad CFX96. GAPDH was used as housekeeping gene. Primer sequences: *MurF-1* Fwd: TGTGCAAGGAACAGAAGAC; Rev: CCAGCATGGAGATGCAGTTA; *Atrogin-1* Fwd: TTGGATGAGAAAAGCGGCAG; Rev: TACAGTATCCATGGCGCTCC; *Klf15* Fwd: AAATGCACTTTCCCAGGCTG; Rev: CGGTGCCTTGACAACTCATC; *Gapdh* Fwd: GGGGCTGGCATTGCTCTCAA; Rev: TTGCTCAGTGTCCTTGCTGGGG.

### RNA Sequencing

To study the corticosterone-induced transcriptome in quadriceps muscle, male and female mice were subcutaneously implanted in the neck region with slow-release pellets containing corticosterone (50 mg corticosterone and 50 mg cholesterol; N=6 per sex) or vehicle (100 mg cholesterol; N=6 per sex) (corticosterone and vehicle pellets were synthesized at Leiden University Medical Center). After 14 days, mice were fasted for 5 hours and killed by cardiac puncture under isoflurane anaesthesia (28). Quadriceps muscle was collected and homogenized in Tripure using a Kimble pellet pestle followed by a phase-separation with chloroform. Total RNA was isolated using the RNeasy kit according to manufacturer’s instructions (Qiagen 74104). RNA quality was ensured (RNA Integrity number > 7.0 and 28/18s ratio > 1.0) using the RNA 6000 Nano kit bioanalyser (Agilent). Stranded mRNA libraries were constructed and 100bp paired-end bulk RNA-sequencing was performed at BGI Genomics (Hong Kong, China) on the DNBseq platform. Over 20 million reads were sequenced per sample. RNA sequencing data has been deposited in NCBI’s Gene Expression Omnibus (GEO series accession number GSE202787).

### RNA Sequencing Data Analysis

The RNA-seq pipeline (version 4.1.0), published as part of BioWDL, was used for read quality control, alignment and quantification. BioWDL contains the main sequencing analysis pipelines and workflows developed at Leiden University Medical Center by the sequencing analysis support core with code being accessible at https://biowdl.github.io/.

Quality control was performed using FastQC and MultiQC. Reads were aligned to Mus Musculus genome version 10 (mm10) using STAR (version 2.7.3a). Tool settings used were: ‘–runThreadN’ ‘4’ ‘–outSAMunmapped’ ‘Within KeepPairs’ ‘–twopassMode’ ‘Basic’. The gene-read quantification was performed using HTSeq-count (version 0.12.4). Tool settings used were: ‘–order’ ‘pos’ ‘–stranded’ ‘reverse’. Uniquely assigned reads were mapped to known genes based on Ensembl release 97 of mm10. HTSeq-count output files were merged into a count matrix per experiment as input for differential gene expression analysis.

DEseq2 (version 1.29.4) was used for normalization of the count data (median of ratio’s method) and identification of differentially expressed genes. For the differential expression analysis, all genes which were expressed in a minimum of four replicates with >20 normalized counts for at least one of the groups were selected. This resulted in 13,049 genes that were included in the analysis. Pair-wise comparisons of groups within experiments were analysed and a false discovery rate adjusted p-value of 0.01 and a log2FC <-1 or >1 was used as a cut-off for detection of differential gene expression. Principal component analysis was performed using DEseq2 and heatmaps of scaled, normalized counts were made with pheatmap (version 1.0.12). Gene ontology (GO) term enrichment analysis was performed with the ViSEAGO package (version 1.4.0), using fisher’s exact test with 0.01 as a significance cut-off.

### Histology and Immunofluorescence Microscopy

Muscles were isolated and frozen in liquid nitrogen-cooled isopentane. Samples were stored at -80˚C until further processing. Gastrocnemius tissue was cryo-sectioned (8 µm thick) using a cryostat (Leica CM3050S). Cryosections were first stained with rabbit anti-laminin (1:100, Abcam) for 3 hours. After washing with PBS/Tween, sections were stained with secondary goat-anti-rabbit antibodies conjugated to Alexa Fluor-647 (1:1000, Abcam). Sections were incubated overnight at 4°C with a mixture of the following fiber-type specific fluorophore-conjugated primary antibodies (Molecular Probes, Life Technologies): BA-D5 conjugated to Alexa Fluor 350 (1:400; type 1), SC-71 conjugated to Alexa Fluor 488 (1:800; type 2B), and BF-F3 conjugated to Alexa Fluor 594 (1:600; type 2A). A Zeiss Axio Observer A1 microscope was used for imaging. Area quantification and representative pictures were acquired *via* ZEN 2 software.

### Image Quantification

Carl Zeiss Image format were converted to multichannel TIFF files, and image processing was performed in Fiji. Mean fluorescence intensity (MFI) was recorded from each myofiber object using a modified Muscle J macro (34). In brief, tissue masks from the laminin staining were created to determine muscle regions for quantification. The masks were then manually corrected, removing technical artifacts such as tissue folds. To improve the myofiber segmentation outputs, a classifier was trained in the *Ilastik* pixel classification algorithm (35). All the masked laminin images were processed followed by myofiber segmentations defined as the region of interest. Mean MFI and geometrical properties were recorded for each myofiber. As the laminin segmentation was automated, non-myofiber objects were removed by implementing a percentile filtering for the pixel-classification on the object boundary, pixel-classification in the interior of the object, cross-sectional area and the circularity values. After the filtering step, MFI values for each of the three MyHC isoforms were scaled per myofiber as previously described (36). MFI values for each vehicle group were normalized as 1 transformed (natural logarithm) and a myofiber-based MFI analysis was carried out in R (version 3.5.1).

### Statistical Analysis

Statistical analyses were performed with SPSS (version 25) and GraphPad Prism version 9.0.1. The following statistical analyses were used: ANOVA with Turkey multi-comparison according to different variables (including 1-way ANOVA for one variable, 2-way ANOVA for two variables) and unpaired Student t-test. All data are presented as means ± SEM.

## Results

### Corticosterone Treatment Induces Muscle Atrophy in Male and Female Mice, but Specifically Decreases Grip Strength in Female Mice

**Figure 1:**
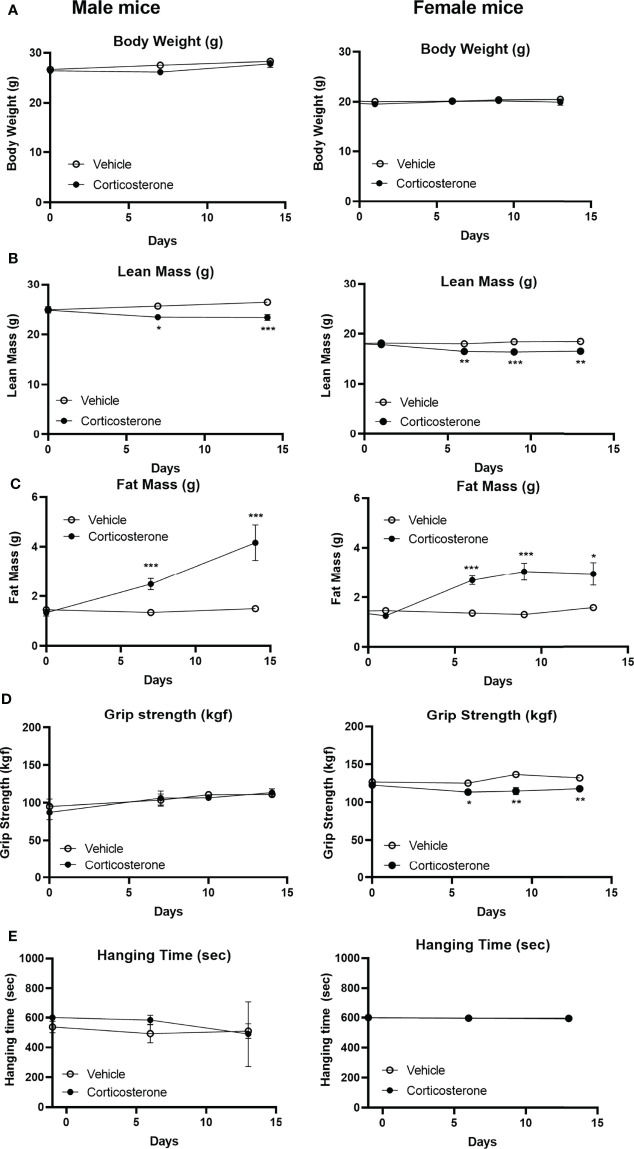
Corticosterone treatment specifically decreases muscle function in female mice. The effect of corticosterone treatment on **(A)** total body mass, **(B)** lean mass, **(C)** fat mass, **(D)** fore limb grip strength and **(E)** grid hanging in male and female C57BL/6J mice. N=8 mice/group. *p<0.05 vs. Vehicle, **p<0.01 vs. Vehicle, ***p<0.001 vs. Vehicle. Statistical significance was calculated using a two-way ANOVA.

**Figure 2:**
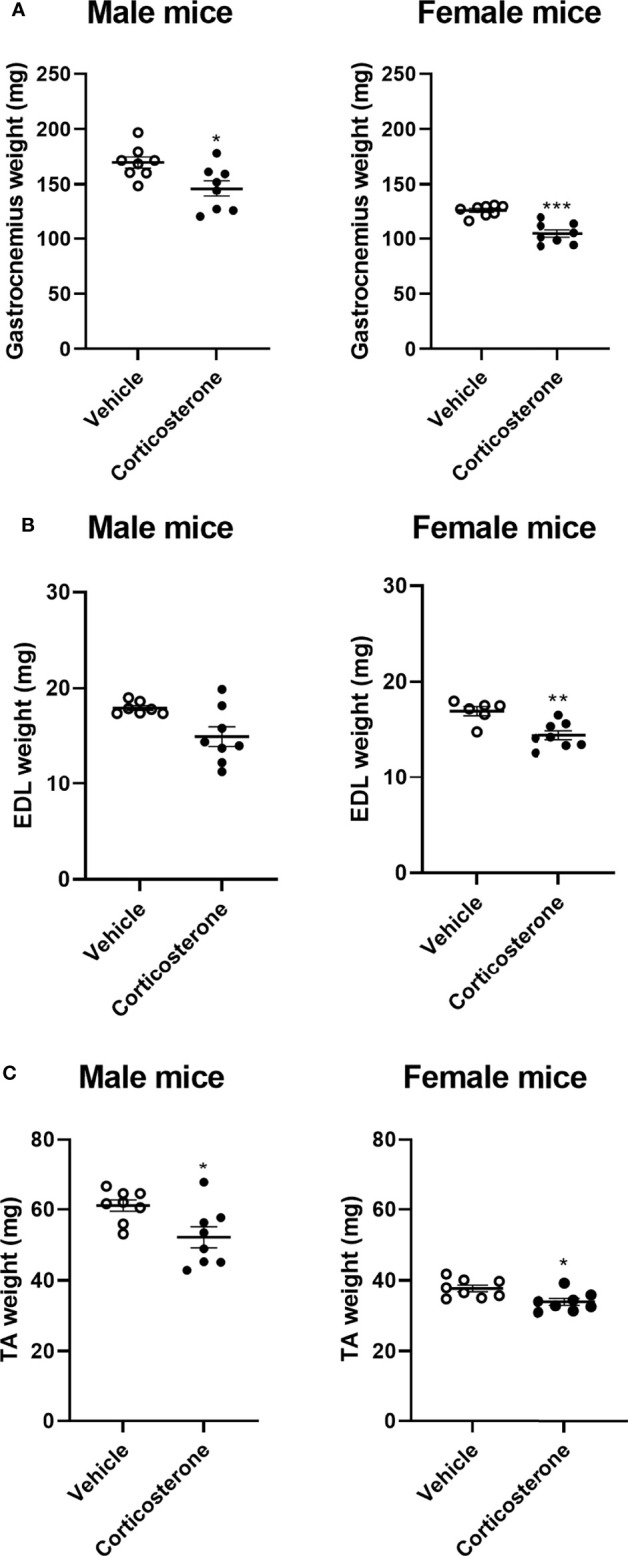
Corticosterone treatment causes muscle atrophy in male and female mice. The effect of corticosterone treatment on tissue weight of **(A)** gastrocnemius, **(B)** extensor digitorum longus (EDL) and **(C)** tibialis anterior (TA) of male and female C57BL/6J mice. N=8 mice/group. *p<0.05 vs. Vehicle, **p<0.01 vs. Vehicle, ***p<0.001 vs. Vehicle. Statistical significance was calculated using an unpaired Student’s t-test.

To identify the effects of corticosterone treatment on muscle function, male and female mice were subcutaneously implanted with a corticosterone or vehicle slow-release pellet. Total body weight was not altered in male and female mice during the two weeks of corticosterone treatment (**Figure 1A**). Corticosterone treatment significantly decreased total lean mass (**Figure 1B**) and increased fat mass in male and female mice (**Figure 1C**). Muscle function was assessed by fore limb grip strength and grid hanging measurements. We found that fore limb grip strength was significantly lower in female mice upon corticosterone treatment, but not in male mice (**Figure 1D**). Grid hanging time was unaffected in both male and female mice, since the majority of mice reached the maximum hanging time of 600 seconds (**Figure 1E**). To investigate muscle atrophy, we assessed weights of the gastrocnemius (**Figure 2A**), extensor digitorum longus (EDL) (**Figure 2B**) and tibialis anterior (TA) (**Figure 2C**). Decreased muscle weights were found for all muscles in both sexes, albeit not significant for male EDL (**Figures 2A–C**). Overall, the results show that corticosterone treatment has sexual dimorphic effects on grip strength but not muscle atrophy.

### Corticosterone Treatment Has Sexually Dimorphic Effects on Transcription in Quadriceps Muscle

**Figure 3:**
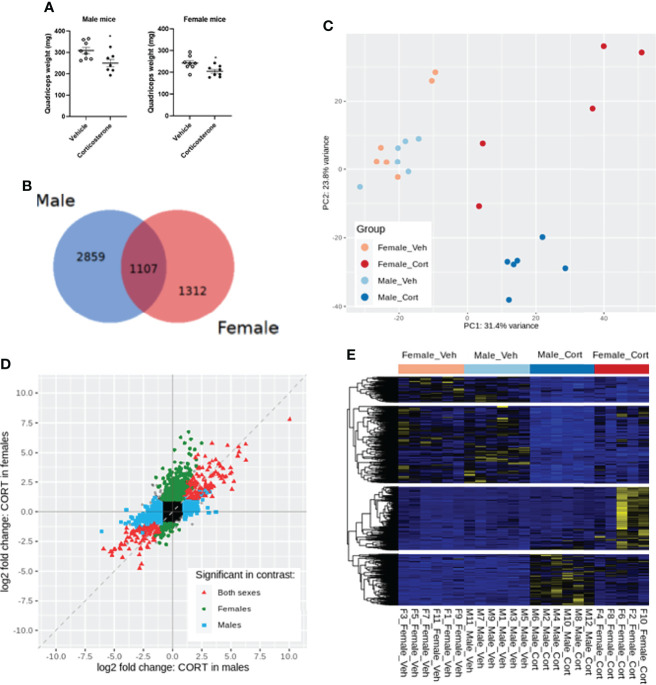
Transcriptome profiling of the quadriceps in male and female mice after corticosterone treatment. **(A)** The effect of corticosterone treatment on muscle weight of the quadriceps. **(B)** Venn-diagram representing sex-specific and shared differentially expressed genes upon corticosterone treatment. **(C)** Principal component analysis of vehicle- and corticosterone-treated male and female C57BL/6J mice. **(D)** Fold change-fold change plot comparing significant changes in corticosterone-treated male and female mice. Male-specific differentially expressed genes are shown in blue, female-specific genes in green, and genes differentially expressed in both sexes in red. **(E)** Heatmap showing all genes regulated by corticosterone. A blue color code represents low expression, a yellow color code high expression. N=6 mice/group for all groups except female-Cort (N=5).

We next investigated the transcriptional effects of corticosterone treatment in the quadriceps, which also showed atrophy (**Figure 3A**). We analysed quadriceps muscle from corticosterone- and vehicle-treated male and female mice by bulk RNA sequencing. When using an adjusted p-value of 0.01, we identified 1107 differentially expressed genes upon corticosterone exposure that were shared between male and female mice, while 2859 genes were specifically regulated in male mice and 1312 genes were specifically regulated in female mice (**Figure 3B**). Of the 1107 shared genes, the expression of 213 genes was significantly different between male and female mice after corticosterone treatment. These differences were predominantly driven by the extent of change in relative expression between male and female mice. For the expression of 11 genes, this differences was explained by an effect in opposite direction (down in female and up in male: *Atmin*, *Setd3*, *Zfp622*, *Eif6* and *Nr1h2*; up in female and down in male: *Dhdds*, *Slc27a4*, *Cyb5b*, *Ctsz*, *Anxa2* and *Adams5*). Principal component analysis showed that biological replicates of corticosterone-treated male mice clustered closely together, while female mice exhibited considerable variation in muscle transcriptome in response to corticosterone exposure (**Figure 3C**). Further scrutiny confirmed the considerable overlap in corticosterone-regulated genes between male and female mice as well as the sex-specific effects (**Figure 3D**). Hierarchical clustering generated four clusters of corticosterone-regulated genes – visualized in a heatmap - comprising of genes similarly regulated between sexes (upper two gene clusters) and sex-specific effects (lower two gene clusters) (**Figure 3E**).

When evaluating expression of specific genes, we first evaluated the expression of genes that encode for the superfamily of nuclear steroid receptors. We found that the *Nr3c1* gene (encoding for the glucocorticoid receptor) was significantly downregulated by corticosterone treatment only in female mice, while *Nr3c2* (mineralocorticoid receptor) was only significantly downregulated in the quadriceps of male mice (**Supplementary Table 1** and **Supplementary Figure 1A**). *Nr3c4* (androgen receptor) was not significantly changed by corticosterone treatment and *Esr1* (estrogen receptor-α) was strongly downregulated in the quadriceps muscle of both male and female mice (**Supplementary Table 1** and **Supplementary Figure 1A**). *Nr3c3* (progesterone receptor) and *Esr2* (estrogen receptor-β) were not detected in quadriceps muscle. We next assessed classical GR target genes, including *Fkbp5, Tsc22d3* (*Gilz*)*, Per1, Sgk1* and *Zbtb16*, as proxies for GR activity. *Fkbp5* was similarly regulated in male and female mice in response to corticosterone, while *Tsc22d3* (*Gilz*)*, Per1* and *Sgk1* were specifically upregulated in male mice (**Supplementary Table 2** and **Supplementary Figure 1B**). In an attempt to better understand the sex-specific effect of corticosterone, we performed a go-term analysis. Analysis of differentially expressed genes after corticosterone treatment (adjusted p-value 0.01 and log2FC of <-1 and >1) showed many pathways that were specifically regulated in male mice, including the muscle atrophy pathway (25, 37), with 37.5% of genes differentially expressed in male mice (p<0.01) and 0% in female mice (**Supplementary Table 3**). Male-specific regulated genes involved in atrophy included *Klf15* and its downstream ubiquitin-ligases *MurF-1* (*Trim63*) and *atrogin-1* (*Fbxo32*) (**Supplementary Table 4** and **Supplementary Figure 1C**). Other factors involved in ubiquitination, including *UBC*, *Ube4b* and *Usp14*, were not influenced by corticosterone exposure in male and female mice. Noteworthy sex differences in the response to corticosterone were the stronger upregulation of the *FoxO1, -3* and -*4* transcription factors in male as compared to female mice. There were no clear effects of corticosterone on several proteasome subunits, and factors related to autophagy were similarly upregulated in male and female mice (*LC3*) or unaffected in both sexes (*Bnip3*) (**Supplementary Table 4** and **Supplementary Figure 1C**). We validated a selection of genes in the quadriceps muscle of male and female mice from the functional cohort. Based on this, it seemed that *Klf15* and *Per1* expression was specifically increased in male mice (p=0.051 and p=0.099), while *Nr3c1* (*Gr*) was specifically decreased in female mice at trend level (p=0.053 in female mice; **Supplementary Figure 2**). Collectively our data show many transcriptional effects are stronger in male quadriceps in response to corticosterone treatment, including genes related in muscle atrophy, in contrast to the functional data in which male and female mice both exhibited muscle atrophy and decreased grip strength was specific to female mice.

### Daily Betamethasone Treatment Decreases Grip Strength and Muscle Atrophy in Both Sexes

**Figure 4:**
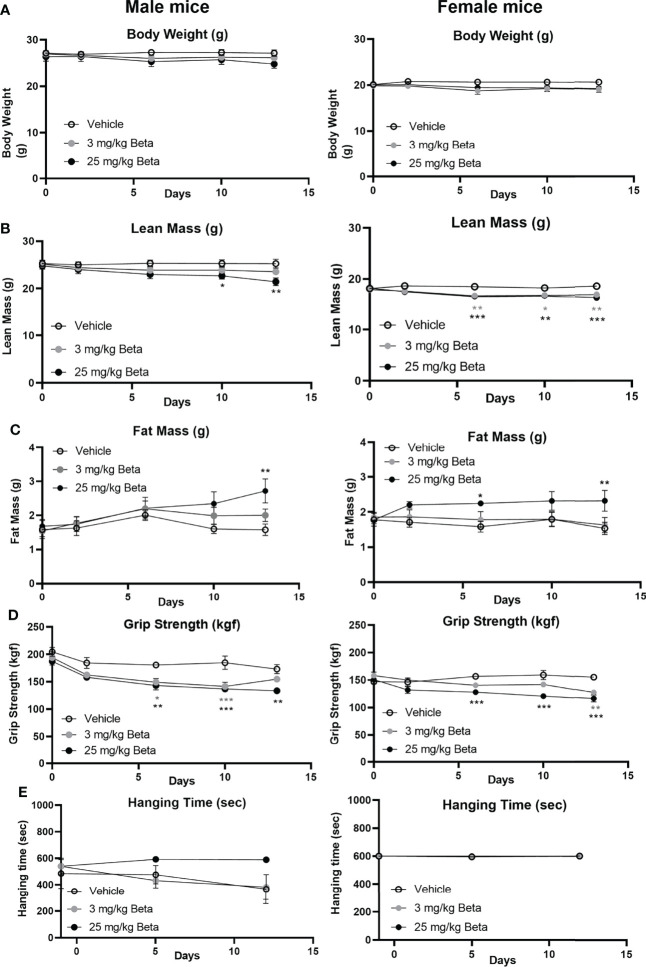
Daily betamethasone treatment decreases muscle function in male and female mice. The effect of daily treatment with 3 or 25 mg/kg betamethasone on **(A)** total body mass, **(B)** lean mass, **(C)** fat mass, **(D)** fore limb grip strength, and **(E)** grid hanging time in male and female C57BL/6J mice. N=4 male mice/group, N=6 female mice/group. *p<0.05 vs. Vehicle, **p<0.01 vs. Vehicle, ***p<0.001 vs. Vehicle. Statistical significance was calculated using a one-way ANOVA.

**Figure 5:**
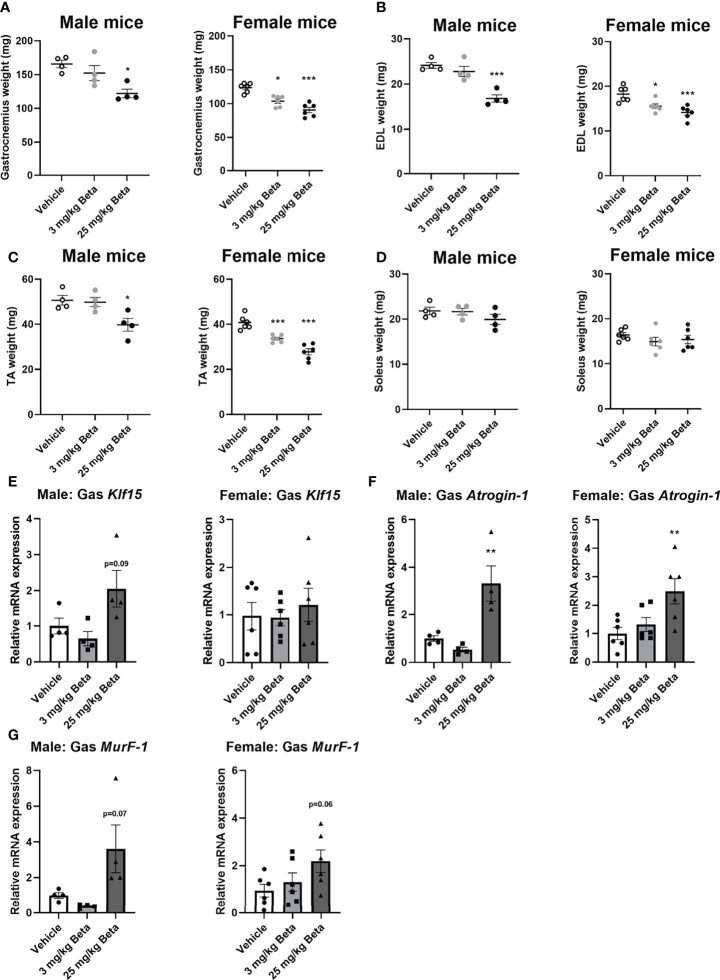
Female mice exhibit muscle atrophy at lower doses of daily betamethasone treatment as compared to male mice. The effect of daily treatment with 3 or 25 mg/kg betamethasone on tissue weight of **(A)** gastrocnemius, **(B)** extensor digitorum longus (EDL), **(C)** tibialis anterior (TA) and **(D)** soleus. The effect of 3 or 25 mg/kg betamethasone treatment on gene expression in gastrocnemius muscle of **(E)** *Klf15*, **(F)** *atrogin-1* and **(G)** *MurF-1*. N=4 male mice/group, N=6 female mice/group. *p<0.05 vs. Vehicle, **p<0.01 vs. Vehicle, ***p<0.001 vs. Vehicle. Statistical significance was calculated using a one-way ANOVA.

**Figure 6:**
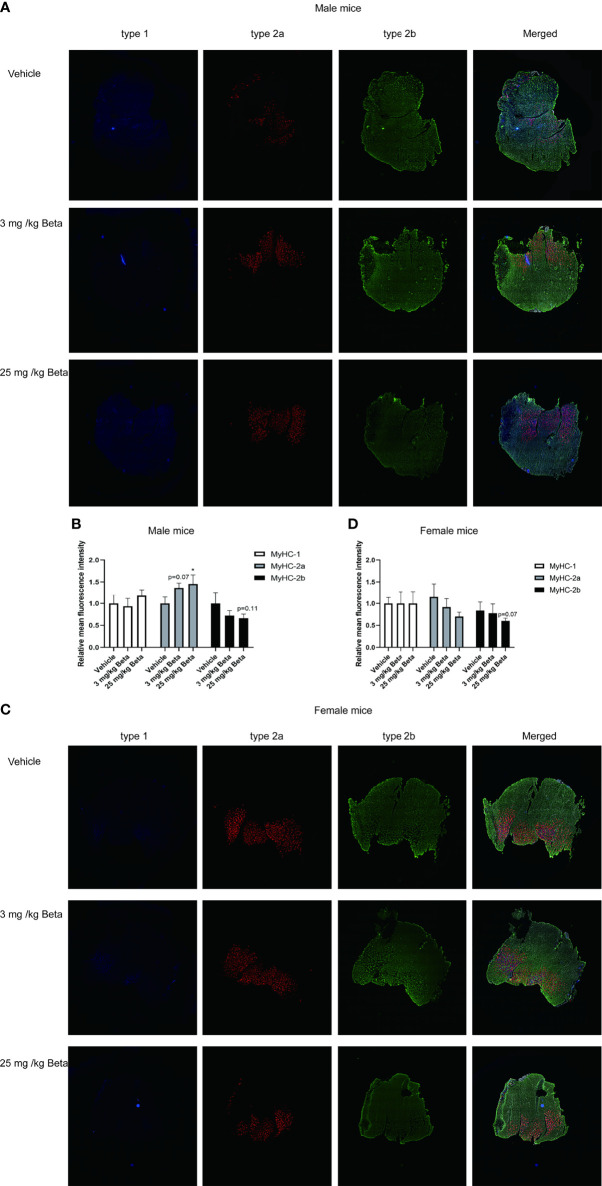
Daily betamethasone treatment increases abundance of type 2A myofibers in male mice. Histological analysis of gastrocnemius muscle for type 1, type 2A and type 2B myofibers. **(A)** Representative images of myofiber staining in gastrocnemius muscle of male mice and **(B)** Relative mean fluorescence intensity for individual myofiber isoforms. N=3 mice/group. **(C)** Representative images of myofiber staining in gastrocnemius muscle of female mice and **(D)** Relative mean fluorescence intensity for individual myofiber isoforms. N=3 mice/group. Type-1=Blue; Type-2a=Red; Type-2b=Green. *p<0.05 vs. Vehicle.

To evaluate the effects of synthetic glucocorticoid treatment on muscle function, male and female mice were injected daily with 3 or 25 mg/kg betamethasone for a period of 2 weeks. In both sexes, body weight was not significantly different after daily betamethasone treatment (**Figure 4A**). Treatment with 25 mg/kg betamethasone significantly decreased lean mass of male mice, while this was observed for both doses in female mice (**Figure 4B**). Fat mass was increased in both male and female mice upon 25 mg/kg betamethasone treatment (**Figure 4C**). In both male and female mice, treatment with 25 mg/kg betamethasone significantly reduced grip strength (**Figure 4D**). However, treatment with 3 mg/kg betamethasone transiently decreased grip strength in male mice, while grip strength in female mice was only decreased after 14 days of treatment (**Figure 4D**). Grid hanging performance was not significantly altered after betamethasone treatment (**Figure 4E**). To investigate the effect of daily betamethasone treatment on muscle atrophy, we analysed different muscles. Gastrocnemius weight of female mice was significantly decreased after 3 and 25 mg/kg betamethasone treatment, whereas in male mice muscle weight only decreased upon 25 mg/kg betamethasone treatment (**Figure 5A**). Similar patterns were observed for the muscle weights of EDL (**Figure 5B**) and TA (**Figure 5C**). In the glucocorticoid-resistant soleus muscle, we did not observe any significant effects of betamethasone treatment on muscle weight (**Figure 5D**). Gene expression analysis of atrophy-related genes in the gastrocnemius muscle revealed *atrogin-1* was significantly upregulated in both male and female mice after 25 mg/kg betamethasone treatment, while *Klf15* and *MurF-1* expression was not significantly altered (**Figures 5E-G**). Female mice showed an upregulation of *atrogin-1* and *MurF-1* in gastrocnemius muscle after 25 mg/kg betamethasone treatment, but not of *Klf15* (**Figures 5F, G**). We next investigated the effect of betamethasone treatment on myofiber composition in the gastrocnemius. As expected, gastrocnemius muscles of vehicle-treated male mice were comprised of relatively few type 1 and type 2A fibers, and primarily type 2B fibers (**Figures 6A, B**). In contrast, vehicle-treated female mice had relatively many type 2A fibers (**Figures 6C, D**). Betamethasone treatment in male mice significantly increased the proportion of type 2A myofibers (**Figure 6B**). In female mice, type 1 and type 2A myofiber composition was unaffected by betamethasone treatment, while type 2B numbers tended to be decreased after daily betamethasone treatment (**Figure 6D**). Collectively, these functional data suggest that female mice exhibit betamethasone-induced muscle atrophy at lower doses as compared to male mice, with similar effects on grip strength between sexes.

### Daily Betamethasone Treatment and Chemical Castration Additively Cause Muscle Atrophy

**Figure 7:**
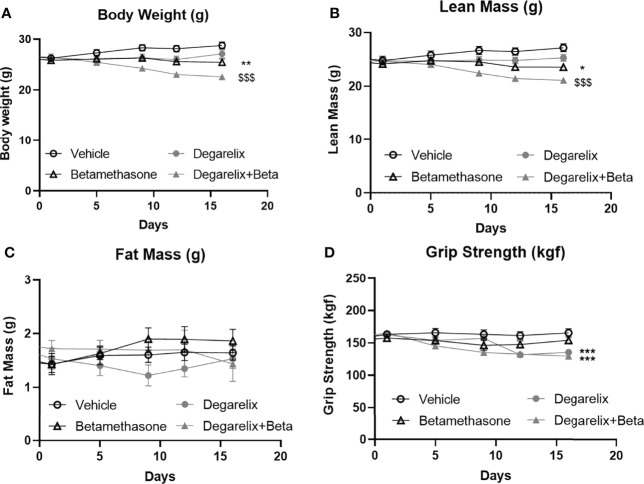
Daily betamethasone treatment reduces total body weight and lean mass in intact and chemically-castrated male mice. The effect of daily treatment with 3 mg/kg betamethasone in chemically-castrated mice and intact mice on **(A)** total body mass, **(B)** lean mass, **(C)** fat mass, and **(D)** fore limb grip strength. N=8 mice/group. *p<0.05 vs. Vehicle, **p<0.01 vs. Vehicle, ***p<0.001 vs. Vehicle, $$$ p<0.001 vs degarelix. Statistical significance was calculated using a two-way ANOVA.

**Figure 8:**
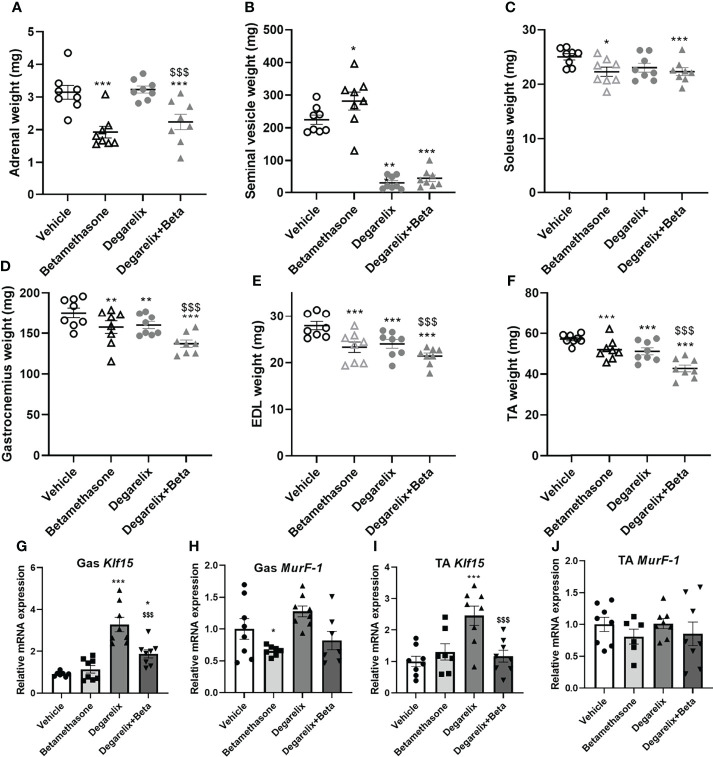
Chemical castration and daily betamethasone treatment additively decrease muscle weight in male mice. The effect of daily treatment with 3 mg/kg betamethasone on intact and chemically-castrated male mice on weight of the **(A)** adrenal gland, **(B)** seminal vesicle, **(C)** soleus, **(D)** gastrocnemius muscle, **(E)** extensor digitorum longus (EDL) and **(F)** tibialis anterior (TA). The effect of daily treatment with 3 mg/kg betamethasone on intact and chemically-castrated male mice on expression of **(G)** *Klf15* in gastrocnemius, **(H)** *MurF-1* in gastrocnemius, **(I)** *Klf15* expression in TA, and **(J)** *MurF-1* expression in TA. N=8 mice/group. *p<0.05 vs. Vehicle, **p<0.01 vs. Vehicle, ***p<0.001 vs. Vehicle, $$$ p<0.001 vs degarelix. Statistical significance was calculated using a two-way ANOVA.

To explore mechanisms underlying male-female differences in glucocorticoid response in muscle function, we investigated potential contribution of androgen signalling in glucocorticoid-induced muscle atrophy (30). To this end, we chemically castrated male mice using the GnRH antagonist degarelix. Intact and chemically-castrated male mice were subsequently injected daily with 3 mg/kg betamethasone or vehicle for 2 weeks. During this treatment period, chemical castration alone did not significantly influence total body weight and lean body mass, but seemed to potentiate the effect of betamethasone treatment on body weight and lean mass (**Figures 7A,B**). Fat mass appeared to transiently decrease upon chemical castration, but was not influenced by betamethasone treatment (**Figure 7C**). Chemical castration on its own decreased fore limb grip strength, and additional betamethasone treatment did not further influence this (**Figure 7D**). Post-mortem analysis showed decreased adrenal weight after betamethasone treatment regardless of the chemical castration (**Figure 8A**). Diminished seminal vesicle weight was observed after chemical castration (**Figure 8B**). Analysis of muscle tissue showed that chemical castration alone significantly decreased gastrocnemius, EDL and TA, but not soleus weight (**Figures 8C–F**). Treatment with 3 mg/kg daily betamethasone reduced gastrocnemius weight in intact male mice, and further reduced muscle weight in chemically-castrated mice (**Figure 8D**). Similar observations were found for EDL and TA muscle weight, for which betamethasone treatment further decreased muscle weight of chemically-castrated mice (**Figure 8E, F**). Gene expression analysis revealed that in gastrocnemius muscle, degarelix treatment induced the expression of *Klf15*, an effect that was significantly lowered by betamethasone treatment (**Figure 8G**). Betamethasone treatment lowered *MurF-1* expression, which was unaffected by chemical castration (**Figure 8H**). Similarly to its effect in gastrocnemius, degarelix treatment induced *Klf15* in tibialis anterior muscle, while *MurF-1* expression was unaltered (**Figures 8I, J**). Collectively, these data suggest that androgen depletion and glucocorticoid treatments both have separate effects on muscle atrophy, and that combined intervention possibly has additive effects.

## Discussion

In this study, we set out to investigate sexual dimorphism in glucocorticoid-induced muscle dysfunction. Muscle dysfunction as a result of elevated glucocorticoid exposure is common in patients with hypercortisolism but is also frequently observed during synthetic glucocorticoid treatment regimens. Although the magnitude of this problem in clinical practice is evident, to our knowledge no studies exist that have addressed sex differences and the role of sex hormones in glucocorticoid-induced muscle dysfunction. We observed that corticosterone treatment caused muscle atrophy in male and female mice to a similar extent at the dose used, based on the analysis of five different skeletal muscles. Despite similar atrophy-inducing effects by corticosterone, only female mice exhibited a decreased grip strength, while male mice were unaffected by this. We performed an extensive transcriptomic analysis of male and female quadriceps muscle after corticosterone exposure in an attempt to capture the similarities and differences between sexes. Overall, we observed more differentially expressed genes in male mice as compared to female mice (2859 male-specific genes versus 1312 female-specific genes after corticosterone treatment). This finding was also evident when analyzing several classic GR-target genes (e.g. *Gilz*, *Per1* and *Sgk1*) that were found to be regulated more strongly in male mice as compared to female mice. It is interesting to note that in contrary to the results above, increased glucocorticoid levels as response to fasting shows greater induction of GR-regulated genes in female gastrocnemius muscle as compared to male (38). The response to glucocorticoid/GR-induced transcription thus appears context-dependent but may also be muscle fiber type-dependent. In corticosterone-treated female mice, we found a large within-group variation in transcriptomic response (as represented in the PCA analysis and heatmap), and this is possibly related to different stages of the estrous cycle at which tissues were collected for which we did not stratify.

Despite the finding that corticosterone had similar atrophy-inducing effects in male and female mice, gene ontology analysis revealed muscle atrophy as one of the main glucocorticoid-induced sexually dimorphic pathways, and indeed male-specific upregulations of atrophy-related genes were found for *Klf15*, *atrogin-1* and *MurF-1*, amongst several others. Despite the stronger transcriptional response to corticosterone in quadriceps muscle of male mice (including genes related to muscle atrophy), this did not yield stronger atrophy-induction in males, and decreased grip strength was even specific for female mice. It is likely that different pathways contribute to muscle atrophy in male and female muscle, and in addition to direct catabolic effects, antagonism of anabolic pathways can also underlie the decreased muscle mass by glucocorticoids. It should be noted that the transcriptomic analysis was performed after 14 days of elevated corticosterone exposure – a timeframe that allows adaptation in tissue response - and thus transcriptional effects after acute corticosterone treatment may differ. Furthermore, the corticosterone dose in the transcriptomic cohort (50 mg) was higher as compared to the functional cohorts (20 mg) and these experiments were performed at different institutes. We also observed that reduced muscle mass does not necessarily influence muscle function (grip strength). However, a potential limitation of our study is that we analyzed muscle weight of the hind limbs, while the functional test evaluated forelimb muscle strength. Nevertheless, given the similar atrophy induction in several muscles, we expect that the observed effects in hind limps are representative for most muscles in the mouse including the forelimbs. A notable exception is the soleus muscle – previously reported to be largely resistant to glucocorticoid-induced atrophy likely attributed to fiber composition mainly consisting of type 1 and 2A fibers and low GR expression levels (25). In our studies the soleus was also largely unaffected by synthetic glucocorticoid treatment in both male and female mice.

For synthetic glucocorticoid treatment with betamethasone, we show that both male and female mice exhibited reduced grip strength, with both doses that were tested in this study. We found that male mice are less sensitive to betamethasone-induced muscle atrophy – and treatment with 25 mg/kg/day was required to induce atrophy in male mice while 3 mg/kg/day betamethasone induced this in female mice. Differences in sensitivity are not explained by GR expression levels, as these were reported to be similar in male and female gastrocnemius muscle (38). Also analysis of atrophy-related gene expression (39) in gastrocnemius muscle did not reveal any noteworthy differences between male and female mice in response to betamethasone treatment. We observed that betamethasone induced a transformation of muscle fiber types in gastrocnemius muscle (17, 40, 41), with increased type 2A and decreased type 2B fiber proportions in male mice. Such a shift of muscle fiber isoforms was previously associated with a reduction of muscle strength (42, 43). It is interesting to note that we observed different effects of corticosterone treatment and betamethasone treatment in male mice, as grip strength in male mice was not influenced by corticosterone treatment while significantly reduced upon betamethasone treatment. These discrepancies between both glucocorticoids may be explained by differences in pharmacokinetic and pharmacodynamic properties, but also by their receptor specificity and corticosteroid-binding globulin binding affinity.

We postulated that sex differences in glucocorticoid effects on muscle may be related to relative androgen levels. Androgens are well-known anabolic factors that are involved in muscle physiology, and increased anabolic signaling may protect from glucocorticoid-induced muscle atrophy and dysfunction. To assess to what extent androgen signaling contributes to the observed sex differences, we chemically castrated male mice using GnRH antagonist degarelix. Androgen depletion on its own strongly reduced forelimb grip strength, but betamethasone treatment did not further influence this. Weight of several muscles was reduced after androgen depletion – likely due to reduced anabolic signaling. In addition, chemical castration of male mice seemed to potentiate the atrophy-inducing effects of low dose betamethasone treatment, but for many muscles both effects were additive rather than synergistic. Our findings thus cannot rule out separate anabolic and catabolic signaling pathways, and do not provide direct evidence for crosstalk between these pathways.

Sex-based differences in skeletal muscle physiology are known in humans, including differences in fiber type prevalence which translates to altered performance, endurance and recovery of skeletal muscles (44). In addition, humans show differences in glucocorticoid response between sexes. In patients with subclinical hypercortisolism, women exhibited lower skeletal muscle mass but men do not (45). Moreover, differential expression of 11β-HSD1 likely influenced local glucocorticoid turnover. Indeed, higher 11β-HSD1 expression in women is associated with reduced grip strength (46). It should be noted that while 11β-HSD1 influences (local) levels of endogenous glucocorticoids, many synthetic glucocorticoids are not a substrate for such enzymatic (in)activation and their activity is thus unlikely to be affected. Also at the receptor level, differences between men and women exist, and polymorphisms in the GR gene were shown to reduce grip strength in male patients with overt hypercortisolism (Cushing’s syndrome) (47). Altogether, many aspects of glucocorticoid response in skeletal muscle are different between men and women. Our study in mice provides suitable models to further investigate the mechanisms that underlie these sex-specific effects, and propose that differential androgen levels may in part contribute to these discrepancies.

## Data Availability Statement

The datasets presented in this study can be found in online repositories. The names of the repository/repositories and accession number(s) can be found below: GEO, GSE202787.

## Ethics Statement

The animal study was reviewed and approved by Leiden University Medical Center animal welfare committee and Erasmus MC animal welfare committee.

## Author Contributions

SL, OM and JK contributed to the conception and design of the study. SL, MiS, TM, MG, MaS, JK performed the experiments in this study. JB performed the analysis of the RNA-sequencing data. JV and KK provided the samples for RNA-sequencing analysis. DV, TA-D and VR performed myofiber stainings and image analysis. AA-R and MP provided technical advice and expertise for the muscle function tests. OM and JK supervised the study. SL and JK wrote the first draft of the manuscript. All authors contributed to manuscript revision, read and approved the submitted version. All authors contributed to the article and approved the submitted version.

## Funding

SL was supported by a Chinese Scholarship Council grant. This study was in part funded by the Leiden University Fund-Mulder Hamelers obtained by JK.

## Conflict of Interest

The authors declare that the research was conducted in the absence of any commercial or financial relationships that could be construed as a potential conflict of interest.

## Publisher’s Note

All claims expressed in this article are solely those of the authors and do not necessarily represent those of their affiliated organizations, or those of the publisher, the editors and the reviewers. Any product that may be evaluated in this article, or claim that may be made by its manufacturer, is not guaranteed or endorsed by the publisher.

## Supplementary Material

Supplementary Figure 1 Quadriceps RNA sequencing data of selected genes. **(A)** Normalized read counts for **(A)** genes encoding for steroid nuclear receptors, **(B)** classical glucocorticoid receptor-target genes, and **(C)** genes included in the gene ontology term muscle atrophy.

Supplementary Figure 2 Quadriceps RT-PCR analysis of selected genes. Relative mRNA expression of *Gr*, *Klf15*, *Fkbp5* and *Per1* in quadriceps muscle of male and female mice after corticosterone treatment (20 mg). N=6-8 per group. Statistical significance was calculated using an unpaired students t-test.
