# Supplementary figures and images for: Sexual Dimorphism in Transcriptional and Functional Glucocorticoid Effects on Mouse Skeletal Muscle
Source: Front Endocrinol (Lausanne). 2022 Jul 11;13:907908. doi: 10.3389/fendo.2022.907908 (PMC9309696; doi:10.3389/fendo.2022.907908)

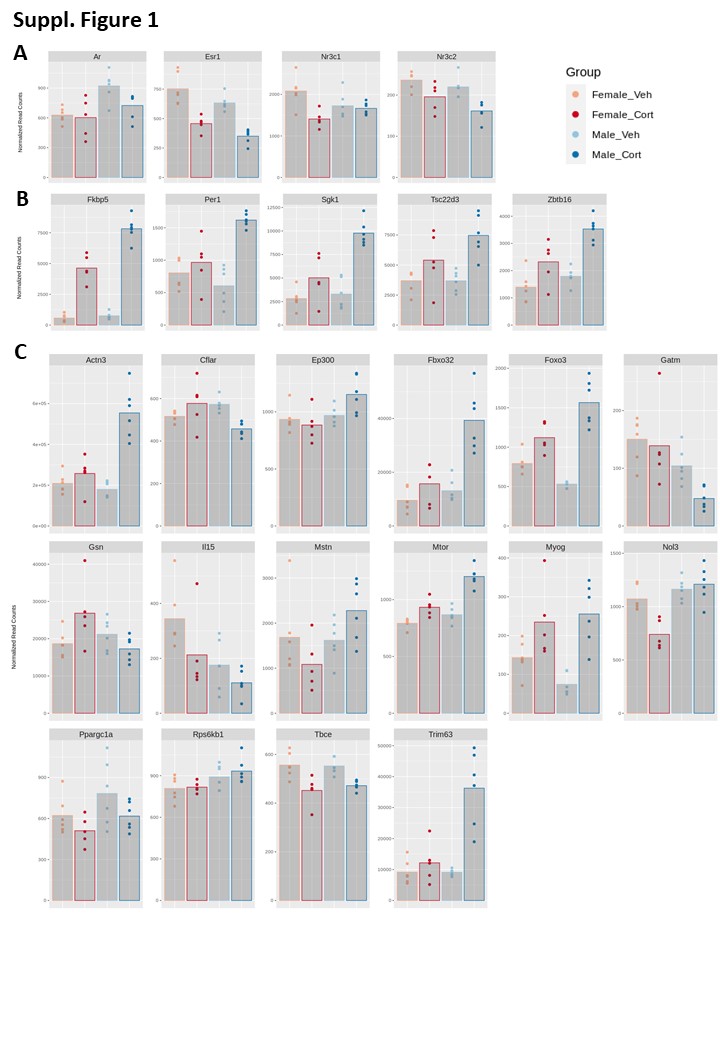

Supplement: Supplementary Figure 1 — Quadriceps RNA sequencing data of selected genes. (A) Normalized read counts for (A) genes encoding for steroid nuclear receptors, (B) classical glucocorticoid receptor-target genes, and (C) genes included in the gene ontology term muscle atrophy. [file Image_1.jpeg]

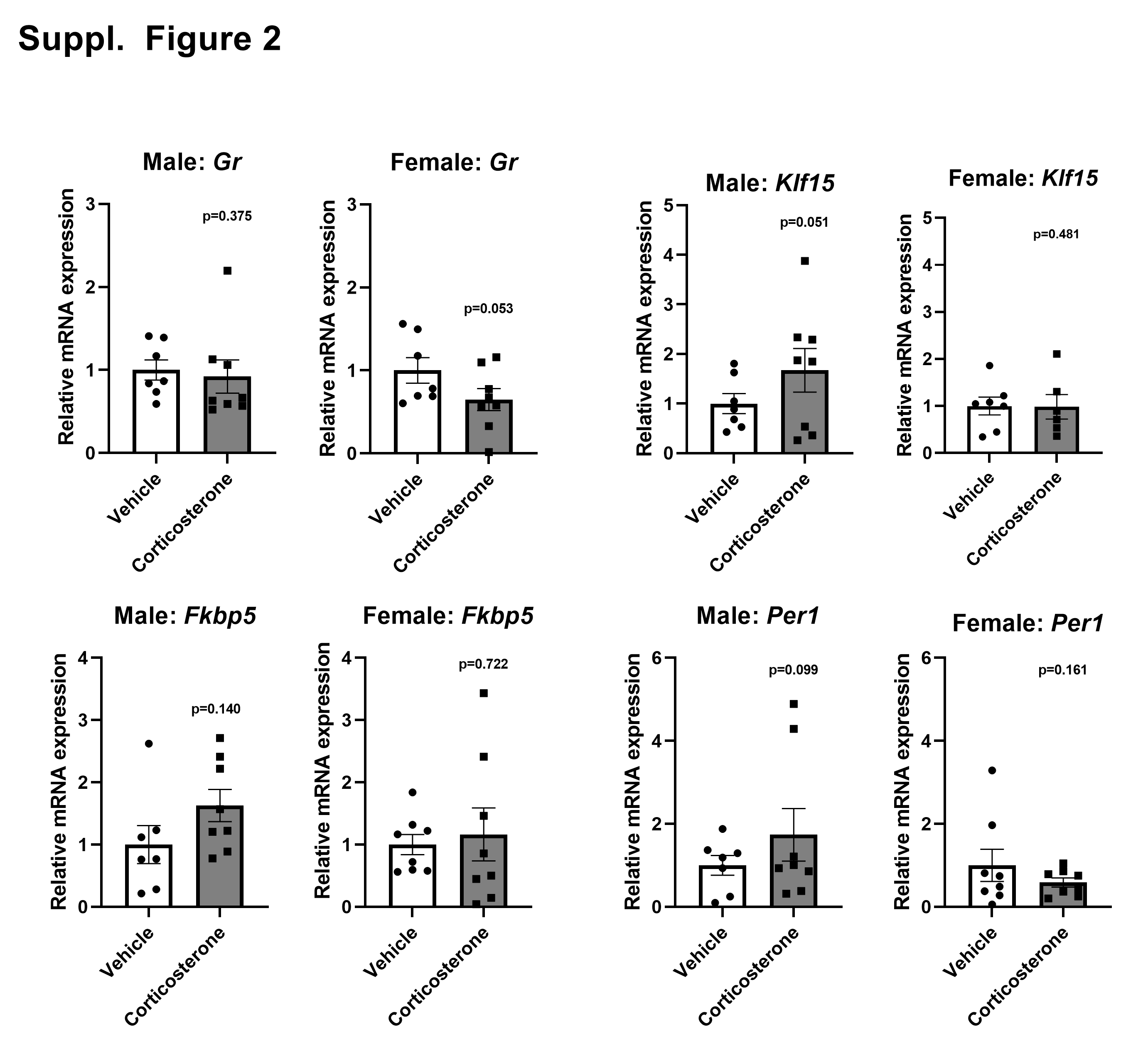

Supplement: Supplementary Figure 2 — Quadriceps RT-PCR analysis of selected genes. Relative mRNA expression of Gr, Klf15, Fkbp5 and Per1 in quadriceps muscle of male and female mice after corticosterone treatment (20 mg). N=6-8 per group. Statistical significance was calculated using an unpaired students t-test. [file Image_2.tif]
